# Preoperative injection of carbon nanoparticles is beneficial to the patients with thyroid papillary carcinoma: From a prospective study of 102 cases

**DOI:** 10.1097/MD.0000000000011364

**Published:** 2018-07-06

**Authors:** Shouyi Yan, Wenxin Zhao, Bo Wang, Liyong Zhang

**Affiliations:** a Department of Thyroid and Vascular Surgery; b Department of General Surgery; c Minimal Invasive Center, Fujian Medical University Union Hospital, Fuzhou, Fujian Province, China.

**Keywords:** carbon nanoparticles, papillary thyroid cancer, parathyroid grand, preoperative injection

## Abstract

**Background:**

More surgeons had noticed the importance of carbon nanoparticles (CNs) in protection of parathyroid grand in the surgery of thyroidectomy and central lymph lode dissection, but paid less attention to the injection time. The purpose of this study was to investigate whether preoperative injection of CNs can improve the dissection of lymph nodes (LNs) and protect parathyroid grand (PG) for the patients with papillary thyroid carcinoma (TC).

**Methods:**

A total of 102 consecutive patients were enrolled into this study from August 2016 to June 2017. All the patients were divided randomly into preoperative group and intraoperative group by the injecting time of the CNs. We compared the patients who had CNs injected into thyroid gland 1 month before surgery with a control group of patients who had CNs injected during the operation. The primary endpoints were operative time, numbers of total LN and metastatic LN, ratio of PG auto-transplantation, parathyroid hormone (PTH) level, and postoperative complications.

**Results:**

We identify 206 PGs and 162 PGs in the preoperative and intraoperative group, respectively, (*P = *.000) and there was low ratio of auto-transplantation in the preoperative group compared with the intraoperative group (39.3% vs 50.62%, *P = *.003). We also found that the PTH level in the preoperative group was higher than that of preoperative group (2.60 ± 1.00 vs 2.19 ± 0.72, *P = *.021), and the operation time in the preoperative group was less than the intraoperative group (60.17 ± 6.28 vs 80.94 ± 7.12, *P = *.000). Meanwhile pathological results revealed 3 PGs of accidental removal occurred in the preoperative group, whereas 9 PGs of accidental removal occurred in the intraoperative group (*P = *.039). Also there was no difference in the numbers of total and metastatic LN in the 2 groups (*P > *.05).

**Conclusion:**

Preoperative injection of CNs was safe, and can help protect PG and reduce the difficulty of operation.

## 1 Introduction

In thyroid surgery, hypoparathyroidism was a common and serious complication, having a serious effect on the patient's life quality especially on the patients with total thyroidectomy (TT) and bilateral central lymph node dissection (BCND).^[1–4]^ The key point of preventing permanent hypoparathyroidism was the identification of PG during the operation,^[5,6]^ it mainly depended on physical recognition of PG by the operator such as color, texture, and surface microvasculature of PG. Meanwhile the identification of PG was closely related to the operative experience of the surgeon.^[7,8]^

Now we have paid much attention in protecting PG to avoid hypocalcaemia during operation. In recent years, a novel type of lymphatic tracer, carbon nanoparticles (CNs) has been applied in the protection of parathyroid glands by staining lymph node into black except the PG, and it could help operator identify the parathyroid glands quickly. More surgeons had noticed the importance of carbon nanoparticles (CNs) in protection of PG during thyroidectomy,^[9,10]^ but there was still a problem of CNs leaking during the operation, which may affect the identification and protection of the PG by staining the thyroid region black accidently. As we know, the use of carbon CNs was usually performed during the operation, but in our medical center we found that preoperative injection of CNs has a better effect by reducing the leakage of CNs in the surgical region. So the aim of the study was to investigate the effects of CNs injected in the different time including preoperation and during operation for the patients with bilateral PTC or CN+ metastasis.

## 2 Material and methods

### 2.1 Patients characteristics

A total of 102 consecutive patients were enrolled into this study from August 2016 to June 2017, and the characteristics of patients were showed in Table 1. All the patients were diagnosed as bilateral PTC or metastatic of lateral LN by preoperative FNA (fine needle aspiration). According to the injective time of CNs, they were divided into 2 groups: preoperative group and intraoperative group. In the preoperative group, CNs were injected into thyroid gland 1 month before the operation, while it was injected during the operation in the intraoperative group. The inclusion criterion was as following: the longest diameter of PTC was <4 cm). The patients were confirmed as bilateral PTC, or confirmed with metastatic of lateral LN by postoperative pathology. Also exclusion criteria were as showing: Postoperative pathology suggests that there were other types of tumors, such as medullary or undifferentiated cancer. Tumor had invaded parathyroid glands during the operation. The patient had a history of thyroid surgery. The age was <16 years. Had an inability to comply with the follow-up. All the patients were randomly divided into 2 groups: preoperative group (n = 54) and intraoperative group (n = 48) according to the injection time of CNs. The randomization was done by using computer-generated random number tables. All the operations were performed by the same surgeon, and all the patients underwent total thyroidectomy plus dissection of central lymph node additionally at least. The study was approved by the ethics committee of Fujian Medical University Union Hospital, and all patients provided an informed consent.

**Table 1:**
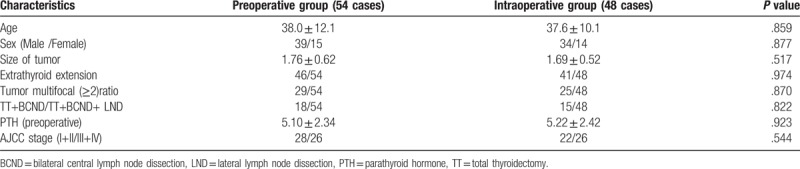
The clinical characteristics of the patients.

### 2.2 Imaging agent for lymphatic vessels: carbon nanoparticles

**Figure 1:**
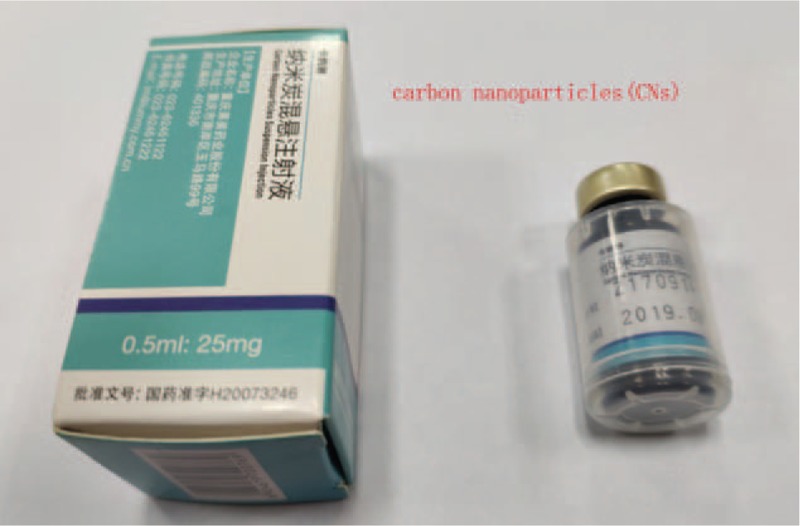
Carbon nanoparticles.

Carbon nanoparticles, Chongqing LaiMei pharmaceutical co. Ltd., production license, the Food and Drug Administration Production Permit No (China) 2007204, registration number: National prescription H20041829 (Fig. 1).

CNs was injected with a stoste into the thyroid tissue. This product was a stable suspension of carbon pellets of 150 nm in diameter. The diameter of carbon pellets is greater than the capillary endothelial cell gap (approximately 20–50 nm) and smaller than the lymphatic capillary endothelial cell gaps (approximately 120–500 nm). A small amount of carbon particles was captured by macrophages, which then entered the lymphatic duct but not the blood circulation. No toxic side effects have been reported for CN in humans.^[11,12]^

### 2.3 Surgical procedure

#### 2.3.1 Injection of CNs one month before the surgery in the group A

Patients were in the position of high shoulder with a pad, and disinfection around the puncture point was prepared before the injection of CNs. Then operator extracted 0.1 mL CNs and injected into the thyroid in 2 spots in each thyroid lobe by ultrasound guided, and confirmed no bleeding in the injection area.

#### 2.3.2 Injection of CNs during the surgery in the group B

**Figure 2:**
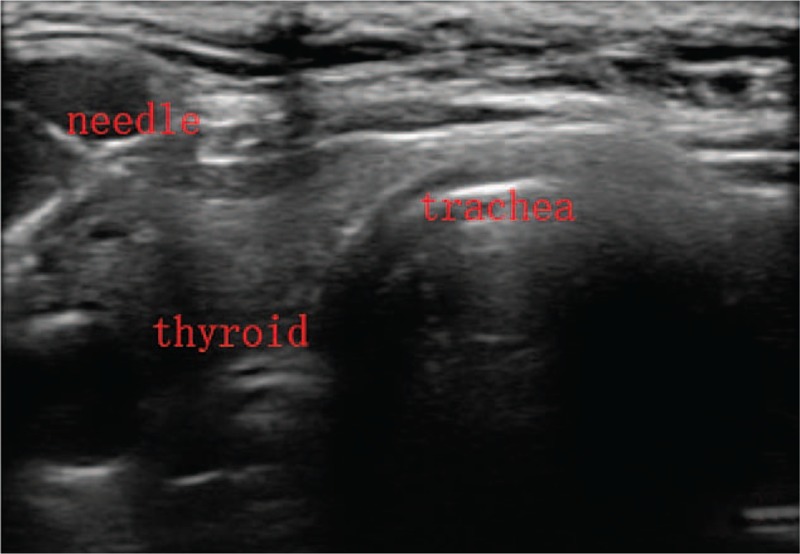
The needle penetrated into normal thyroid tissue for CNs injection. CNs = carbon nanoparticles.

**Figure 3:**
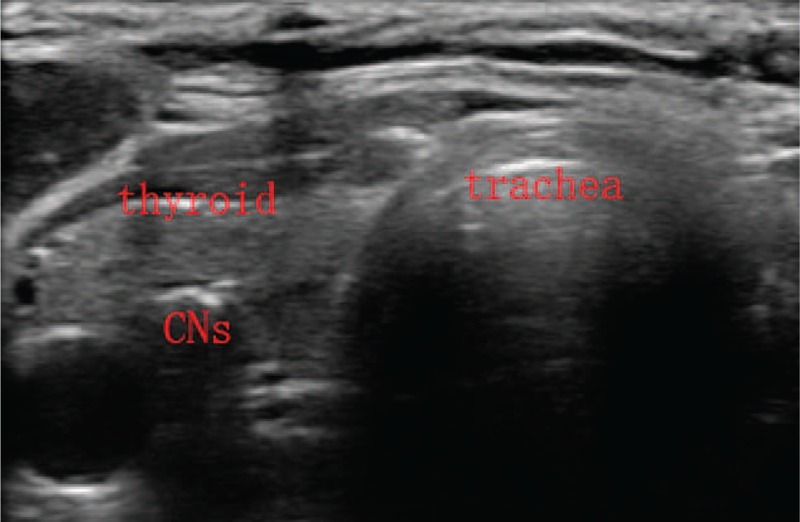
The needle retreated with some negative pressure after CNs injection, then we can found the CNs with a high echo in the thyroid tissue. CNs = carbon nanoparticles.

When the strap muscles retracted, the front ipsilateral thyroid was revealed, and residual of the lobe were kept intact for reducing damage of lymphatic network around the thyroid. Then 0.1 mL CNs was injected into the both sides of normal thyroid tissue by using a syringe (1 mL). Two spots were needed for each lobe and the total volume of injected CNs was 0.4 mL. Meanwhile we had to notice that the needle of the syringe with CNs should be inserted deeply and CNs should not be injected into the blood vessel. At last, needle was retreated gently with negative pressure to prevent CNs leakage (Figs. 2 and 3).

#### 2.3.3 Selection of surgical methods

The patients without metastatic of lateral LN were treated with the total thyroidectomy and dissection of bilateral central lymph node, and the patients with metastatic of lateral LN were treated with the total thyroidectomy, bilateral central lymph node dissection, and lateral lymph node dissection (LND).

#### 2.3.4 Identifying and protection of parathyroid glands during the operation

We know that the anatomic location of the superior PG is constant on the dorsal aspect of the upper thyroid lobes at the level of the inferior border of the cricoid cartilage, but the location of inferior PG is variable due to their embryologic relationship to the thymus.^[13–15]^ The key point of PG identifying is to observe the staining (nonstained black), blood supply (micro vessel on the surface of PG), texture (the hardness is between the fat and the lymph nodes), color (light brown), and the neighboring relationship (usually connected with the thymus). When looking for the PG during the operation, we should attempt to preserve it in situ. If the PG was lack of blood supply or completely free with thymus, it would be transplanted into the forearm in time. Also if PG cannot be found during operation, we should identify it in the resected specimens according to the dyeing of tissue and physical characteristics of PG, which could be transplanted into the forearm in time.

### 2.4 Follow-up and postoperative treatment of hypoparathyroidism

The level of parathyroid hormones (PTHs) would be tested in 3 time points including 2 weeks, 2 months, and 6 months after the operation. Postoperative hypoparathyroidism was defined as that the level of parathyroid hormones (PTHs) was <1.3 mmol/L after 6 months. Calcium supplementation was not routinely administered to patients, but calcium and vitamin D were routinely prescribed to patients with symptomatic hypoparathyroidism until the level of PGH recovered to normal. Intravenous substitution of calcium was not a routine unless serious symptomatic hypocalcaemia was present. Routine treatment of levothyroxine was for all patients after operation. And the time of follow-up was 36–42 months in the all patients.

### 2.5 Data collection

General characteristics, intraoperative factors, pathologic examination, the number of lymph nodes and metastatic lymph nodes in the resected specimens, and postoperative complications were collected retrospectively in the hospital. PG of accidental removal was defined as not finding PG during operation (including careful inspection of the resected specimens) but finding it in the final pathological examination. The seventh edition of American Joint Committee on Cancer (AJCC) staging was used for all the recruited patients. The primary endpoints were operative time, numbers of total LN and metastatic LN, ratio of PG auto-transplantation, PTH level, and postoperative complications.

### 2.6 Statistical analysis

Statistical analysis was performed by SPSS 17.0, Chicago, IL. All values are presented as mean ± standard deviation. A *T*-test or Chi-square test was used to determine statistical significance, requiring *P* < .05 was considered to be statistically significant.

## 3 Results

### 3.1 Patents characteristics

The clinical characteristics of the patients in the 2 groups were summarized in Table 1. PTC was confirmed by postoperative pathology for all patients. There were no significant differences between 2 groups in terms of age (*P = *.859), sex (*P = *.877), size of tumor (*P = *.517), tumor multifocal (*P = *.870), extrathyroid extension (*P = *.974) and TT/TT+LND (*P = *.822), and AJCC stage (*P = *.544).

### 3.2 Identifying and protecting of PG

We could find most of PG concluding conservation in situ and transplantation in the arm, but also miss some inferior PG in the surgery. All the accidentally resected PG in the 2 groups was the inferior one. Pathological results revealed that 3 PGs of accidental removal occurred in the preoperative group, whereas 9 PGs occurred in the intraoperative group. The difference was statistically significant (*P = *.039).

We identify 206 PGs and 162 PGs in the preoperative group and intraoperative group, respectively, there was a significant difference between the 2 groups (*P = *.000) (Table 2). Meanwhile, there was low ratio of auto-transplantation in the preoperative group compared with the intraoperative group (39.3% vs 50.62%), which also is a significant difference (*P = *.003). We also found that the PTH level in the preoperative group was higher than that of intraoperative group (2.60 ± 1.00 vs 2.19 ± 0.72), with a significant difference (*P = *.021).

**Table 2:**
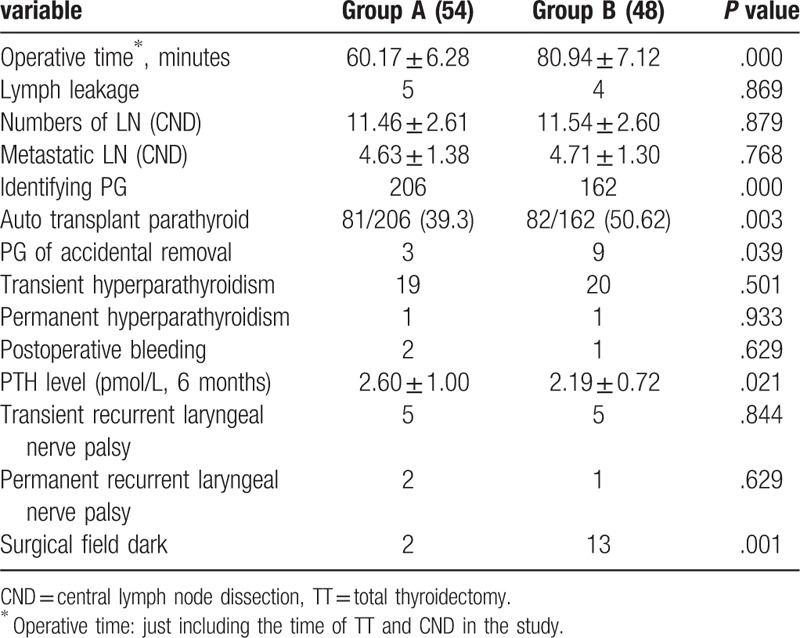
Comparing of variable in the 2 groups.

### 3.3 Central lymph node dissection (CND) in the preoperative and intraoperative group

For the reason that not all the patients were diagnosed with metastatic of lateral LN, we just list and compare the numbers of central LN in this study. All the patients underwent dissection of central lymph node. And there was no statistically significant difference between the 2 groups in the aspect of total LN and metastasis LN (*P = *.879, *P = *.768).

### 3.4 Side effects and operative complications

**Figure 4:**
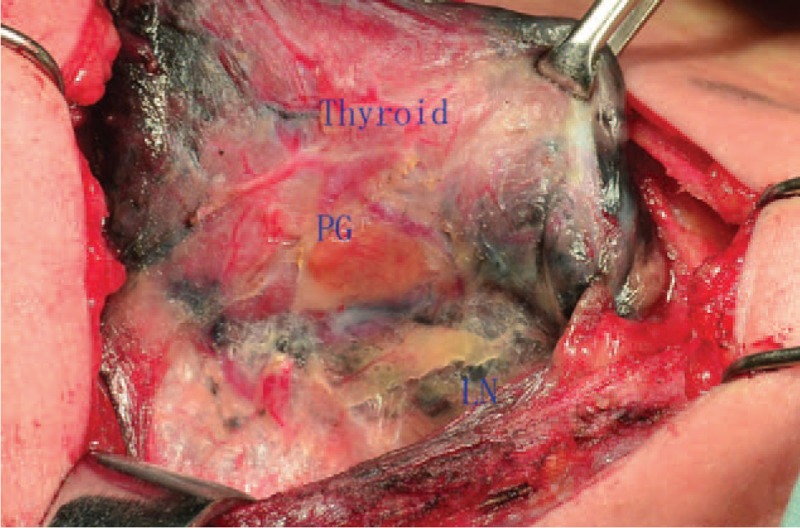
Thyroid and LN were stained dark by the CNs except the parathyroid gland. CNs = carbon nanoparticles, LN = lymph node.

**Figure 5:**
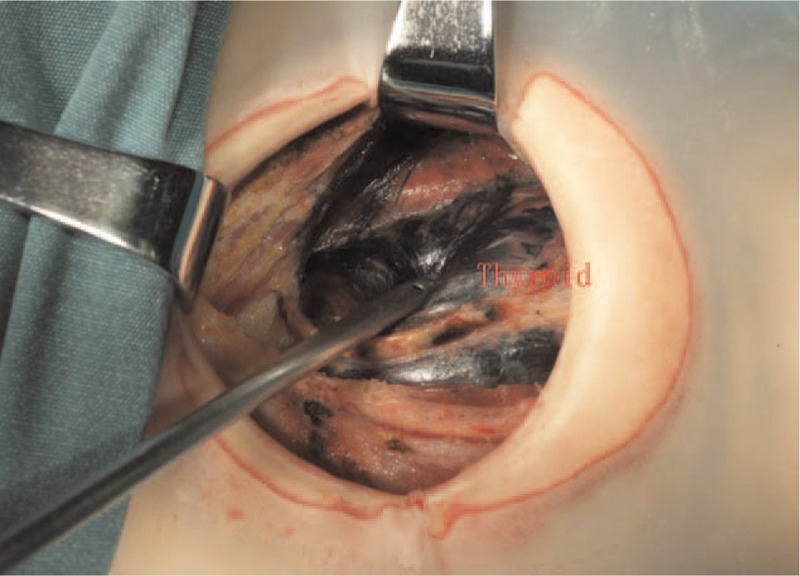
CNs leaked out of the thyroid gland and stained surgical field dark. CNs = carbon nanoparticles.

No obvious systemic toxicity occurred to all patients in the perioperative period of surgery. However in the intraoperative group we found 13 patients were in the phenomenon that CNs leaked out of the thyroid gland and stained surgical field dark, increasing the difficulty of the operation. But there were only 2 patients found with CNs leaking in the preoperative group (Figs. 4 and 5). Meanwhile, we found that the operation time in the preoperative group was less than that of intraoperative group (60.17 ± 6.28 vs 80.94 ± 7.12), There was a significant difference between the 2 groups (*P = *.000).

There were some patients with the complications including postoperative lymph leakage (9/102), postoperative bleeding (3/102), transient hypoparathyroidism (39/102), permanent hypoparathyroidism (2/102), transient recurrent laryngeal nerve palsy (10/102), and permanent recurrent laryngeal nerve palsy (3/102) in this study. But there was no statistical difference in the 2 groups (*P < *.05). The patients with postoperative lymph leakage recovered with conservative treatment in the hospital. The patients of transient hypoparathyroidism did not show the low-calcium symptoms with treatment and the PTH level recovered to normal after 6 months, except 2 patients with permanent hypoparathyroidism, who continued to be followed up. Also there were 3 patients with vocal cord dyskinesia according to electronic laryngoscope after 6 months.

## 4 Discussion

Total thyroidectomy plus central neck dissection (CND) was premise for treating patient with bilateral PTC or CN metastasis.^[16]^ More surgeons had paid much attention in protecting PG to prevent permanent hypoparathyroidism which was the most common complication can seriously affect patients’ life quality.^[17]^ We also know that the precondition of PG protection was the rapid identification during the operation, while the identification of PG was affected by various factors such as obesity, fatty degeneration, bleeding, and so on. A variety of techniques including the double-phase SPECT images, methylene blue had been used to locate and identify PG, but the results have been reported without obvious clinical effectiveness in protection of PG.^[18,19]^

Now the question on how to locate and identify PG quickly would be solved by CNs, Which plays an important role in identifying of PG.^[20,21]^ This product is a stable suspension of carbon pellets of 150 nm in diameter, captured by macrophages, and then particles enters the lymphatic vessels and accumulates in the thyroid and lymph nodes, thus thyroid glands and lymph nodes are stained black except the PG. As many papers report, CNs have been applied to more accurately guide the dissection of lymph nodes and protect the function of PG during thyroidectomy and central neck dissection.^[22]^

More surgeons usually injected the CNs during the operation, and had noticed the phenomenon of CNs leaking out in the surgery. If happening, it would seriously affect the resection of thyroid and dissection of CND, also impact the protection of the PG. Even without the happening of CNs leaking, during operation we also found much exudation which was caused by the CNs. So far, there were no reports about preoperative injection of CNs by ultrasound-guided for the patients with thyroidectomy. In our study, injection of CNs was performed 1 month before operation and the clinic effect was perfect in our result. We found 13 patients were in the situation that CNs leaked out of the thyroid gland and stained operative region dark in the intraoperative group, while it was only 2 patients in the preoperative group. The objective result was that the operation time in the preoperative group was less than the intraoperative group. Meanwhile we identify 206 PGs and 162 PGs in the preoperative group and intraoperative group respectively, there was a significant difference between the 2 groups (*P = *.000). Pathological results revealed that 3 PGs of accidental removal occurred in the preoperative group, whereas 9 PGs occurred in the intraoperative group. The explanation for this was that CNs were easy to leak out of thyroid in the process of separation by destroying the lymph-vessel and thyroid capsule in the intraoperative group, staining the surgical region black and affecting the identifying of PG, and the problem of CNs leaking could not be solved by the compression of thyroid puncture site just for 5 minutes. But as time went on, the influence caused by CNs leaking can be slowly alleviated after one month in the preoperative group. We guessed that time interval between CNs injection and operation may play an important role in eliminating the CNs of intertissue gap and alleviating inflammation caused by CNs leaking. But the theoretical hypothesis we proposed mentioned above could not be supported by the experimental data in this study.

We also found an interesting phenomenon that there was low ratio of auto-transplantation in the preoperative group compared with the intraoperative group (39.3% vs 50.62%, *P = *.003), and the PTH level in the preoperative group was higher than that of preoperative group (2.60 ± 1.00 vs 2.19 ± 0.72, *P = *.021). The reason for that may be as follows: blood supply of PG was too small to be injury in the operation of TT and BCND, especial for the patients with CNs leaking in the intraoperative group, if preserving PG in situ was impossible; we should transplant the PG into the forearm timely. Even auto-transplantation of PG had been proved to be effective, but it would loss of part function,^[23]^ so it may be concluded that the more transplantation of PG, the more loss of its part function.

Since inferior PG had a more variable location, it was hard to find the inferior PG especially for the patients with oldness and obesity. Also if parathyroid glands cannot be found during operation, we should identify parathyroid glands in the resected specimens according to the dyeing of tissue and physical characteristics of parathyroid glands, which could be transplanted into the forearm in time. The result that the entire 12 accidentally resected PGs in the study were the inferior ones accords with the point in our study; the reasons for that are as follows: For the patients with CNs leaking, the surface of inferior PG turns to be dark, and hard to be distinguished from the adipose tissue. The black-stained central surgical region would affect the identifying of inferior PG according to the physical characteristics of parathyroid glands. The reasons mentioned above may explain the difference of PG accidental removal numbers in the 2 groups.

Similar to other studies,^[24,25]^ our study also found that CNs could help detect lymph nodes, and increase numbers of metastatic lymph nodes in the 2 groups, but there was no significant difference in the numbers of total LN and metastatic LN for the reason that CND was performed according to the requirements of the guideline.^[26]^ So CNs could be used to achieve a more radical effect of central neck dissection in both groups, but cannot be affected by the injection time of CNs.

Based on the results from this study, the use of CN was safe^[27]^ and simple before the surgery and there was no obvious systemic toxicity observed in these patients during and after operations. Meanwhile, ultrasound-guided CNs injection was an easy procedure and could be used in every hospital where they can perform the fine-needle aspiration. In our study, it was safe and no occurrences of severe complications were observed. Patients who underwent this procedure did not have any side effects such as uncomfortable feelings, bleeding, and inflammation, which was in accordance with other reports.^[28]^ But during the injection of CNs, we should notice the following points: we had to be sure that the needle should not be stained black to prevent the skin from being stained black. If happening, it would be catastrophic, and difficult to eliminate. We should retreat needle with some negative pressure after injecting CNs into the thyroid, in order to prevent the tissue gap from being stained black. Because the technique was an invasive, we also had to pay much attention to aseptic concept. We should also notice the contraindication of preoperative injection such as treatment with anticoagulation drugs and dysfunction of coagulation.

There were some problems in this article. Firstly, we should consider more time point of preoperative injecting for optimization use of CNs, maybe the injection time of 2 weeks before surgery was better in helping decrease patient's waiting time and alleviate anxiety which should be test in the further study. Meanwhile, preoperative injection of CNs would increase doctors’ daily workload, and need operator spend much time to explain the importance of CNs preoperative injecting, but once the operator master the skill, it will take only some minutes to finish this job. Secondly, although some meaningful results have been obtained, the number of cases was still relatively small. Thirdly, in this study we use CNs stoste but not CNs diluent, which was also the focus of the next step of the study in order to research the clinic effect of CNs.

## 5 Conclusion

Preoperative injection of CNs was a safe and effective method and the procedure was easy to implement. Also it could help us get better clinical effect including aspects of protecting the PG and decreasing the difficulty of surgery. So CNs preoperative injection may be a good choice for the patients with bilateral PTC or CN metastasis.

## Author contributions

**Conceptualization:** Wenxin Zhao.

**Data curation:** Bo Wang.

**Investigation:** Liyong Zhang.

**Methodology:** Wenxin Zhao, Liyong Zhang.

**Project administration:** Shouyi yan.

**Resources:** Wenxin Zhao.

**Software:** Shouyi Yan.

**Writing – original draft:** Shouyi Yan.

**Writing – review & editing:** Shouyi Yan.
